# Third *Borrelia* Species in White-footed Mice

**DOI:** 10.3201/eid1107.041355

**Published:** 2005-07

**Authors:** Jonas Bunikis, Alan G. Barbour

**Affiliations:** * University of California Irvine, Irvine, California, USA

**Keywords:** zoonosis, Lyme disease, Relapsing fever, Borrelia burgdorferi, reservoir mouse, vector tick, Ixodes

**To the Editor:** The white-footed mouse, *Peromyscus leucopus*, is a natural reservoir host of several pathogens, including *Borrelia burgdorferi*, an agent of Lyme borreliosis (LB) (1). *B. burgdorferi* spirochetes are transmitted in the mouse population by *Ixodes scapularis* ticks. This tick vector also bears *B. miyamotoi*, a sister species to the relapsing fever group of spirochetes (2,3). *B. miyamotoi* infects *P. leucopus* in the laboratory (2), but the role of this mouse as a reservoir was not known. Here we report that *P. leucopus* is a reservoir for *B. miyamotoi* in nature and, in addition, that this mouse is host for a third, hitherto unknown, species of *Borrelia*.

In a recent study of a 9-hectare site in a mixed hardwood forest in eastern Connecticut, we found that ≈35% of *I. scapularis* nymphs were infected with *B. burgdorferi* and ≈6% were infected with *B. miyamotoi* (4). For that study of a field vaccine we also collected blood from *P. leucopus* mice captured from June to early September of 2001. DNA was extracted from the blood and then subjected to quantitative polymerase chain reaction (PCR) assay for the presence of *B. burgdorferi* as described (4). In the present study, we analyzed the extracts of 556 blood samples from 298 mice from the nonvaccine control grids by a multiplex, quantitative real-time PCR for 16S rDNA that discriminated between *B. burgdorferi* and *B. miyamotoi* at the site (4). Sixty-nine (12%) of the samples were positive for *B. burgdorferi* and 36 (6%) were positive for *B. miyamotoi*; 5 (0.9%) of the samples were positive for both species. In infected mice, the mean number of *B. miyamotoi* cells per milliliter of blood was 251 (95% confidence limits of 126–631), 5-fold greater than that of *B. burgdorferi* at 50 cells/mL (40–63).

A standard PCR assay of the blood samples with primers for the 16S–23S rDNA intergenic spacer (IGS) was performed as described by Bunikis et al. (5); results suggested the presence of a third species of *Borrelia* among the blood samples of the mice. A uniquely sized amplicon of ≈350 bp was observed in the reactions of 6 of 100 samples that were positive for *B. burgdorferi* and or *B. miyamotoi* by 16S PCR, and of 2 of 31 randomly selected samples that were negative for both *B. burgdorferi* and *B. miyamotoi* (p = 0.3 by 2-sided exact chi-square test).

Samples with the 350-bp amplicon were further investigated by PCR assay with *Borrelia* genus-specific primers for the 16S rRNA gene (rDNA), as described by Barbour et al. (6). The resultant ≈830-bp PCR product from these samples was directly sequenced on a Beckman 3000CEQ automated sequencer (5). The 788-bp sequence was aligned with sequences of other *Borrelia* species representing the LB and relapsing fever clades, and phylogenetic analysis was conducted. The accompanying Figure shows that the new species clusters with the monophyletic relapsing fever group of species rather than with the LB group species. However, the new spirochete is distinct from all other known *Borrelia* spp. with an available 16S rDNA sequence in the GenBank database. Its partial 16S rDNA sequence differed by 3.3% to 4.2% from 9 LB group species and 2.4% to 3.4% from 15 relapsing fever group species. For comparison, intragroup sequence differences were ≤1.9%. On this basis, as well as the finding of partial IGS sequences (GenBank accession nos. AY668955 and AY668956) that were unique among all *Borrelia* spp. studied to date (3,5), we propose that this is a new species of *Borrelia*, provisionally named *Borrelia davisii* in honor of Gordon E. Davis for his contributions to *Borrelia* research and taxonomy.

**Figure:**
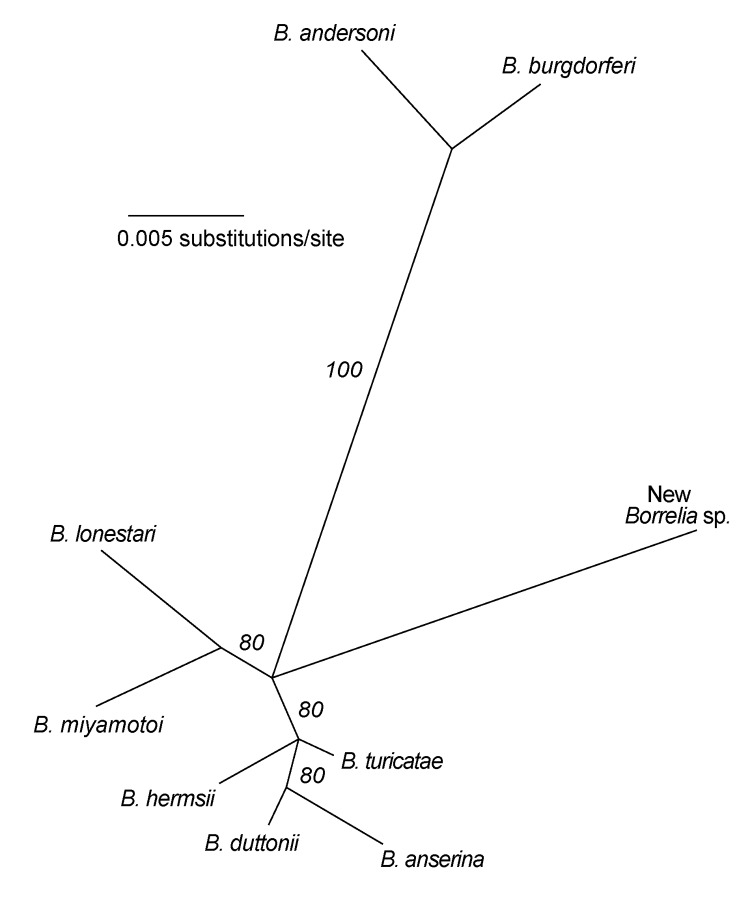
Unrooted maximum-likelihood phylogram for partial 16S rRNA gene sequences of selected Borrelia species, including a novel Borrelia organism, and representing Lyme borreliosis and relapsing fever groups. Sequence alignment corresponded to positions 1138 to 1924 of B. burgdorferi rRNA gene cluster (GenBank accession no. U03396). Maximum likelihood settings for version 4.10b of PAUP* (http://paup.csit.fsu.edu) for equally weighted characters corresponded to Hasegawa-Kishino-Yano model with an empirical estimate of transition/transversion ratio = 7. Support for clades was evaluated by 25 bootstrap replications by using branch-and-bound search, and values >50% are indicated along branches. Sequences (with GenBank accession nos.) used in the analysis were the following: B. andersoni (L46688), B. miyamotoi (D45192), B. lonestari (U23211), B. hermsii (U42292), B. turicatae (U42299), B. duttonii (U28503), B. anserina (U42284), and new Borrelia species (AY536513).

While the new species was detected in 8 of 131 *P. leucopus* blood samples by using PCR for the IGS, the assays for this organism in the DNA extracts of 282 *I. scapularis* nymphs (4) from the same geographic site were uniformly negative (p = 0.0003, 2-sided Fisher exact test). This finding suggests that the new spirochete has another vector. The only other documented tick species that has been found feeding in small numbers on *P. leucopus* in Connecticut is *Dermacentor variabilis* (7). Holden et al. reported the presence of *Borrelia* in *D. variabilis* ticks in California by using PCR with genus-specific primers, but the species in these ticks was not identified by sequencing (8).

Although how *B. miyamotoi* and *B. davisii* affect the health of humans and other animals remain to be determined, our finding of 3 *Borrelia* species with overlapping life cycles in the same host in the same area shows that the ecology of *Borrelia* is more complex than was imagined. The presence of species other than *B. burgdorferi* in a major reservoir will have to be considered in future surveys and interventions.
